# Change in alcohol outlet density and alcohol-related harm to population health (CHALICE)

**DOI:** 10.1186/1471-2458-12-428

**Published:** 2012-06-12

**Authors:** David Fone, Frank Dunstan, James White, Chris Webster, Sarah Rodgers, Shin Lee, Narushige Shiode, Scott Orford, Alison Weightman, Iain Brennan, Vas Sivarajasingam, Jennifer Morgan, Richard Fry, Ronan Lyons

**Affiliations:** 1 Institute of Primary Care & Public Health, School of Medicine, Cardiff University, Heath Park, Cardiff, CF14 4YS, UK; 2 Centre for the Development and Evaluation of Complex Interventions for Public Health Improvement, School of Medicine, Cardiff University, Heath Park, Cardiff, CF14 4YS, UK; 3 School of City and Regional Planning, Cardiff University, Glamorgan Building, King Edward VII Avenue, Cardiff, CF10 3WA, UK; 4 Health Information Research Unit, School of Medicine, Swansea University, Swansea, SA2 8PP, UK; 5 Wales Institute of Social and Economic Research, Data and Methods (WISERD), Cardiff University, 46 Park Place, Cardiff, CF10 3BB, UK; 6 Support Unit for Research Evidence, Information Services, Cardiff University, Heath Park, Cardiff, CF14 4YS, UK; 7 Department of Social Sciences, University of Hull, Cottingham Road, Hull, HU6 7RX, UK; 8 School of Dentistry, Cardiff University, Heath Park, Cardiff, CF14 4XY, UK

**Keywords:** Alcohol, Outlet density, Alcohol-related harm, Anonymised record-linkage, Multilevel analysis, Spatial analysis

## Abstract

**Background:**

Excess alcohol consumption has serious adverse effects on health and violence-related harm. In the UK around 37% of men and 29% of women drink to excess and 20% and 13% report binge drinking. The potential impact on population health from a reduction in consumption is considerable. One proposed method to reduce consumption is to reduce availability through controls on alcohol outlet density. In this study we investigate the impact of a change in the density of alcohol outlets on alcohol consumption and alcohol-related harms to health in the community.

**Methods/Design:**

A natural experiment of the effect of change in outlet density between 2005–09, in Wales, UK; population 2.4 million aged 16 years and over. Data on outlets are held by the 22 local authorities in Wales under The Licensing Act 2003.

The study outcomes are change in (1) alcohol consumption using data from annual Welsh Health Surveys, (2) alcohol-related hospital admissions using the Patient Episode Database for Wales, (3) Accident & Emergency department attendances between midnight–6am, and (4) alcohol-related violent crime against the person, using Police data.

The data will be anonymously record-linked within the Secure Anonymised Information Linkage Databank at individual and 2001 Census Lower Super Output Area levels. New methods of network analysis will be used to estimate outlet density. Longitudinal statistical analysis will use (1) multilevel ordinal models of consumption and logistic models of admissions and Accident & Emergency attendance as a function of change in individual outlet exposure, adjusting for confounding variables, and (2) spatial models of the change in counts/rates of each outcome measure and outlet density. We will assess the impact on health inequalities and will correct for population migration.

**Discussion:**

This inter-disciplinary study requires expertise in epidemiology and public health, health informatics, medical statistics, geographical information science, and research into alcohol-related violence. Information governance requirements for the use of record-linked data have been approved together with formal data access agreements for the use of the Welsh Health Survey and Police data.

The dissemination strategy will include policy makers in national and local government. Public engagement will be through the Clinical Research Collaboration-Cymru "Involving People" network, which will provide input into the implementation of the research.

## Background

Excess alcohol consumption has many adverse effects on health including liver cirrhosis [1], cancer of the oral cavity, pharynx, larynx, oesophagus, liver [2] and breast [3], high blood pressure [4] and stroke [5]. There is also an increased risk of harm resulting from violence including homicide [6], suicide [7], road traffic accidents [8], domestic violence [9], and attendance at Emergency Departments and Minor Injuries Units for treatment of violence-related injuries [10].

Binge drinking is typically defined as consuming double the guideline limits in a single day during the previous week [11]. It is an increasing problem, particularly rising in young women [12], and is associated with anti-social behaviour [13] and around one-half of all violent crimes in the UK [14,15]. Binge drinking places an acute burden on the NHS as around 20–40% of all people presenting to accident and emergency departments are intoxicated, increasing to 80% after 12am [13]. Epidemiological evidence also suggests that binge drinking may reduce the benefits of moderate alcohol consumption on coronary heart disease risk [16,17].

Recent data show that around 37% of men and 29% of women exceed current UK guidelines for safe levels of alcohol consumption (defined in women as > 3 units per day; men > 4 units per day) and 20% of men and 13% of women binge drink (defined in women as > 6 units per day; men > 8 units per day) [18]. Given the wide range of harm resulting from this substantial level of excess consumption, the potential impact on health at the population level from a reduction in consumption is considerable. One of the principal evidence-based policies recently recommended by the British Medical Association to reduce alcohol consumption is to reduce easy access to alcohol through controls on hours of sale and outlet density [19,20]. In this study we focus on alcohol availability measured as outlet density in response to the National Institute for Health Research (NIHR) commissioning brief PHR no. 09/3007.

### Outlet density and alcohol-related harm

A recent systematic review of 44 cross-sectional studies found that the most frequently investigated alcohol-related harm was violent crime, especially assault [21]. The majority of studies (36/44) were from the USA. High outlet densities were associated with a higher rate of assault, self-reported injuries, car crashes, and high rates of pedestrian collisions. Higher outlet density was also shown to be associated with increased domestic violence and child abuse.

Very few studies in the review investigated associations between outlet density and non-injury health outcomes; two studies suggested high outlet densities were associated with an increased rate of sexually transmitted infections (STI) and two out of three studies found an association with high levels of consumption [21]. Of two additional American studies not included in the review, one found an association between higher outlet density and increased alcohol-related hospital admissions in San Diego County, USA [22], and the second study, set in California and Louisiana, found an association with increased self-reported liver disease, STI, and violence [23].

### Change in outlet density and alcohol-related harm

A recent review article, which included papers published up to 2006, summarised the effects of a change in outlet density resulting from three groups of studies: interrupted time series; natural experiments from alcohol bans, and changes in licensing arrangements. None were set in the UK. Many of these studies were old and few accounted for spatial autocorrelation in their analyses (i.e. the lack of independence of proximal small-areas). This review did not include any methodological assessment of the measurement of outlet density. The overall conclusion was that an increase in outlet density was associated with increased consumption and inter-personal violence, but the evidence was weaker for an association with motor vehicle crashes [24].

A further ten studies have investigated associations between change in outlet density and a limited range of outcomes, namely violence [25-31], STI’s [32,33] and suicide [34].

A six-year longitudinal study of change in outlet density set in California found that increases in bar and off-licensed outlet densities were positively related with an increase in the rate of violence, defined as a hospital admission with an overnight stay with ICD-9 ‘E-code’ E960-969 for injuries resulting from inter-personal violence, which are not alcohol-specific [25]. Using the same dataset a second paper reported that change in each measure of outlet density was positively related to likely alcohol-related accident rates, using Police-recorded crashes judged to include alcohol, and hospital admissions resulting from motor vehicle crashes identified from ICD-9 codes E810-E825 (which again are not alcohol-specific) [26]. A smaller study of alcohol-related crashes two years before and after closure of drive-up alcohol outlets in New Mexico reported a non-significant association [27].

A time series analysis of Norwegian data from 1960 to 1995 found outlet density was significantly associated with violent crime [28]. A study set in Melbourne found that an increase in the number of outlets over a nine-year period was positively related to an increase in numbers of violent assaults recorded by the Police as taking place between 8pm and 6am (used as a proxy for alcohol-related assault for which specific data were not recorded) [29]. The fixed effects analyses, which controlled for spatial autocorrelation, modelled numbers of assaults and outlets rather than rates or density. This analysis further categorised postcodes into five clusters, based on socio-demographic factors, to investigate more detailed relationships and found the strongest associations in central and inner-city suburbs.

A study set in Los Angeles reported that a reduction in alcohol availability within census tracts (resulting from a riot in 1992) was associated with a significant reduction in gonococcal infection rates [32], partially mediated by neighbourhood social capital [33]. A significant reduction was also found for violent assaults [30] but a more sophisticated statistical analysis published one year later suggested that the association reported for violent assaults was no longer significant after using a different statistical modelling method [31]. The tenth study, using the same Californian dataset, found that changes in bar and off-premise outlet densities were positively related, and restaurant densities negatively related, to attempted and completed suicide rates [34].

### Outlet density, neighbourhood deprivation and population migration

Two US papers investigated the association between outlet density and deprivation. Higher outlet densities were found in more deprived areas but paradoxically levels of consumption were highest in less deprived areas, suggesting that the mismatch between supply and demand could result in alcohol-related harm being disproportionately higher in people living in deprived neighbourhoods in proximity to alcohol outlets [35,36]. A cross-sectional study set in Glasgow, UK, found that the spatial association between outlet density and deprivation did not vary systematically, highlighting the importance of local context in the design of future studies [37]. Although it is clear from published research that selective population migration may account for a spurious widening of health inequalities over time [38,39], there are no studies which explicitly investigate this possibility for alcohol-related harm outcomes.

### Measurement of outlet density

In the longitudinal studies, outlet density was estimated simply as the number of outlets per resident population in seven of the eight studies which estimated a density [25,27-30,33,34]. One study modelled the number of outlets with the resident population as an explanatory variable [29]. Three studies estimated density using the number of road-miles [30-32]. One of these also used the geographical area as the denominator and reported no difference between the methods [30].

Although most cross-sectional papers used resident population as the denominator, one study set in California noted that there is no standard for measurement of outlet density and estimated outlet density using several different measures for the denominator, including resident population, geographical area (e.g. square mileage), and other more spatial measures of exposure to outlets including Euclidean distance from home to nearest outlet, and number of outlets per 0.5 mile ‘buffer zone’ i.e. the geographical area defined by 10–15 minutes walking time [35]. In this analysis there was little difference in the modelled associations between all of these measures. A New Zealand study used 10 minutes of travelling time by car to define a neighbourhood area, with their density measure as the number of outlets within each neighbourhood [36]. The only published UK research, from Glasgow, estimated outlet density using two different measures: outlets per 1000 residents and the mean network distance from the centroid of each small-area of interest to the nearest outlet [37].

### Primary research question

Overall, there is cross-sectional evidence from mainly American studies to suggest that higher outlet density is associated with alcohol-related harm, particularly involving violence. The evidence from longitudinal studies for associations between harm and a change in outlet density is less well established. Many methodological questions remain over the best way to measure outlet density and how to model the data. Little is known on the effect of a change in outlet density on inequalities in alcohol-related health and the role of population migration. Our primary research question for this study is:

What is the impact of a change in the density of alcohol outlets on alcohol consumption and alcohol-related harms to health in the community?

Our secondary research questions are:

1. Does a health selection effect from population migration at small-area level explain any observed associations between outlet density and alcohol-related harm?

2. What effect does change in outlet density have on population inequalities in alcohol-related health?

## Methods/Design

### Setting

The setting is Wales, UK, total population 3 million; population 2.4 million aged 16 years and over (2001 Census); with 22 local authorities (the administrative areas of local government).

### Design

The intervention we propose to evaluate is the change in the availability of alcohol, measured as outlet density. This complex intervention takes the form of a natural experiment as is common in public health contexts [40], for example in changes to policy on the availability of alcohol. These are not amenable to randomisation but have a natural non-random variability in geographical location and time. Different local authorities have different policies and we will obtain a comprehensive list of these policies and their variations, for example a specific policy for handling applications for licences in areas where there is a danger of saturation. These variations in local policy will be associated with increases and decreases in outlet densities and we plan to contrast the effects of different local policies in our exposure measure and hence in the study outcomes.

We propose four studies**:**

Study 1: Primary outcome: alcohol consumption.

A longitudinal analysis of change in levels of alcohol consumption using data from the 75 000 people aged 16 years and over who anonymously responded to the five annual Welsh Health Surveys carried out between 2005 and 2009 [41]. Consumption will be categorised as an ordinal scale of no alcohol, below sensible limit, above limit, and binge drinking using the Department of Health definition 2007 [42].

Study 2 Secondary outcome: hospital admissions.

A longitudinal analysis of five years of hospital in-patient data for residents of Wales aged 16 years and over using the Patient Episode Database for Wales (PEDW), 2005–09. This includes hospital admissions for alcohol-related causes (around 13 000/year) [43].

Study 3 Secondary outcome: Accident & Emergency attendances.

A longitudinal analysis of residents of Wales aged 16 years and over attending an A&E department at night (midnight–6am, around 100 000/year) as a proxy for alcohol-related injury between 2009 and 2011.

Study 4 Secondary outcome: alcohol-related violent crime.

A longitudinal analysis of five years of Police data on alcohol-related violent crime against the person in Wales, 2005 to 2009 (around 47 000/year).

### Data sources

#### Use of existing record-linked datasets: the Secure Anonymised Information Linkage databank

The Secure Anonymised Information Linkage (SAIL) databank, held and managed within the Health Information Research Unit (HIRU) at Swansea University, contains health, social and education data on three million residents of Wales and currently includes thirteen datasets containing nearly one billion records [44,45]. The smallest geographical area for which data are already linked and may be released is the 2001 Census Lower Super Output Area (LSOA) [46]. These areas vary in size but contain an average of 1500 people, with the constraint of a minimum population of 1000. There are 1896 LSOAs in Wales.

### Welsh Demographic Service (WDS)

The WDS dataset held by NHS Wales Informatics Service (NWIS), the NHS organisation in Wales mandated to hold personally identifiable data, contains addresses for all individuals who register with a General Practitioner. Dates for each update of the address record are held, thereby providing durations of residency for several different homes and the ability to link to local environment exposures at each. Demographic data may be used to create population sub-groups based on age, gender and location for the required date or duration. The WDS contains address information linked anonymously at the individual level (the anonymised linking field, ALF) which is the primary key variable for record-linkage. Using a split-file technique, NWIS supplies ALFs for the whole population of Wales to the SAIL databank [44,45].

### Residential Anonymised Linking Fields

A particular strength of the SAIL system is the development of the system for anonymising all households in Wales and linking household-level data from local authorities and others with individual health-related data whilst protecting anonymity, using Individual Anonymised Linking Fields (ALF) and Residential Anonymised Linking Fields (RALF). Address data are matched at NWIS where identifiable addresses are replaced with RALFs [47]. The residence-based metrics are then fully incorporated into the SAIL databank by linking RALFs to ALFs.

### Environment Geographic Information System

In parallel, but separate from the SAIL databank, is the Environment Geographic Information System (eGIS). The eGIS contains a number of datasets obtained from Great Britain’s mapping agency - the Ordnance Survey. These data include the Ordnance Survey Address Base Premium [48], which contains point data for all residences and businesses, both past, present and future for Wales. The Ordnance Survey MasterMap Integrated Transportation Network dataset [49] provides detailed road and footpath information and the Ordnance Survey Points of Interest database contains places that can be visited, categorised by types, such as pubs, clubs or restaurants [50]. These datasets have typically been surveyed to a spatial accuracy of ± 1-2m, providing high resolution for spatial analysis.

### Alcohol outlets

There are 22 local authorities in Wales who are responsible under The Licensing Act 2003 for maintaining public registers of Premises and Personal Licence holders, available annually from 2005. Under The Act these data are available for public inspection in council offices. Each authority keeps a database (electronic or hard-copy) of the name and address of each premise with the full unit postcode. All 22 local authorities have agreed to supply the data for this study although some authorities do not distinguish between on- and off-sales.

Using the postcode we can georeference each outlet to within any geographical boundary using standard GIS techniques. These postcodes have been validated by the local authorities and we will check each postcode to the address using the Ordnance Survey Address Base Premium [48]. In this study the basic geographical unit for small-area analyses will be the 2001 Census LSOA.

### Estimation of outlet density

Our literature review found four main approaches to estimate outlet density: (1) number of outlets per capita in an administrative unit or geographical area, (2) number of outlets per network miles per geographical area, (3) number of outlets per geographical area defined by walking or drive time (a ‘buffer zone’), and (4) closest Euclidian distance from each subject’s home to an outlet.

We wish to estimate a new measure of outlet density which includes a more realistic and robust measure of accessibility than previously published methods. We will estimate a summary statistic for distance to all outlets from each residence within a defined buffer zone. The measures of distance calculated will be network distances and travel times which will more accurately measure exposure in comparison to Euclidian distances.

We therefore propose a spatial methodology to estimate outlet density using the unique advantages of the WDS population data within the SAIL databank:

(1) We will calculate the sum (or mean) network distance for each individual to each outlet within a meaningful network range (10 minutes walk and 10 minutes drive-time) of each residence (RALF), using the service area tool in ArcGIS Network Analyst module, together with Ordnance Survey MasterMap data and point location facility data in the eGIS [49,50]. The individual-level measure of outlet density exposure will be a function (e.g. reciprocal) of the total (mean) distance;

(2) We will derive the LSOA density by averaging the values for all individual residents in the LSOA.

The primary analysis will be for all alcohol outlets but we will also estimate outlet densities for on and off-sales separately for those authorities who can supply this categorisation.

### Primary outcome: Welsh Health Survey data

Data on consumption will be used from the five annual consecutive Welsh Health Surveys 2005–09 [41]. Participants were asked to state the highest number of units they had drunk on any one day in the previous seven days, using a standard prompt to convert different types and quantities of alcoholic drinks into units (2005–07) or by reporting measures of consumption translated into units by the dataset coders (2008–09). The classification of units into ordinal categories of consumption used in the Welsh Health Survey dataset is based on the Department of Health definitions (Table 1) [42].

**Table 1:** Categorisation of alcohol consumption in the Welsh Health Surveys 2005-09

**Category**	**Maximum units drunk on any day in the last week**
None	Did not drink in the last seven days
Sensible	Men drinking no more than 4 units, women no more than 3 units
Above guidelines, less than binge	Men drinking more than 4 and up to and including 8 units, women more than 3 and up to and including 6 units
Binge	Men drinking more than 8 units, women more than 6 units

Source: References [42].

### Secondary outcomes

#### Hospital admissions

The SAIL databank includes the complete PEDW database, which records all inpatient and day case admissions for residents of Wales. The pseudonymisation process results in the encryption of a unique, unidentifiable ID that enables a patient-based analysis rather than an admissions-based analysis to be carried out. Each hospital spell has an attached ICD-10 diagnosis code in one of 14 positions: primary, subsidiary, and 3^rd^ to 14th position. The Office for National Statistics has published a list of ICD-10 codes to define causes of death which are exclusively alcohol-related i.e. not just where alcohol could be a contributory aetiological factor [51]. There are several other published classifications of ‘exclusively’ alcohol-related conditions with considerable overlap between them [52]. The differences from the ONS definition largely relate to non-fatal conditions excluded from the ONS cause of death list. In addition are the external causes of morbidity and mortality codes for motor vehicle crashes and injuries resulting from inter-personal violence. However these codes are not alcohol-specific. In the analysis we will define alcohol-related as the combined causes of death and condition codes and we will perform secondary analyses including ICD-10 ‘External’ codes for the motor vehicle crashes and injuries resulting from inter-personal violence.

Each PEDW record is linked to the LSOA of residence to which a deprivation score, such as a quintile, of the Welsh Index of Multiple Deprivation 2005 [53], has been attached in SAIL. We can then attribute admissions to an LSOA and deprivation quintile for the analysis.

### Accident & emergency department data

The Accident & Emergency Department Data Set (EDDS) is a new dataset within HIRU/SAIL and the first wave of data for the 13 Accident & Emergency (A&E) Departments in Wales for 2009 has been cleaned and validated. Data for subsequent years are entered into SAIL as it is received, currently on a monthly basis. Attendances are recorded for all injuries with the date and time of day. There is no definitive uniformly applied code for alcohol-related attendances in this dataset and so we will use attendance at night (midnight to 6am) as a proxy for alcohol-related as the majority of injury attendances at night (particularly aged 16–39) are likely to be alcohol related. These attendances will be linked to RALFs and hence to anonymised network measures for the analysis. Using ALFs/RALFs we will also trace attendees who are subsequently admitted to hospital (by linking to PEDW) and exclude all those who do not have an alcohol-related ICD-10 code.

The numbers are sufficient for a spatial analysis: there were 101 908 night time attendances at the 13 A&E departments in Wales in the financial year 2009/10. The highest numbers were on Friday to Saturday and Saturday to Sunday nights (15 862, 17 098) compared to weekdays (weekday range 13 374 to 14 232; weekly mean 14 558, standard deviation 1386).

### Police data

Police forces record electronically all incidents reported to them. Incidents of violence against the person are detailed according to incident type (e.g. assault occasioning actual bodily harm), the injury incurred, incident location and alcohol use. These incidents are recorded at the premises, address or street location level. Eastings and Northings locations are recorded, facilitating georeferencing into LSOAs, although the accuracy of these coordinates has not been evaluated. Data sharing agreements between Cardiff University and the four police forces in Wales are now in place. Our work with police data on an exploratory trial of a disorder reduction intervention found the data to be sufficiently robust for research purposes [54]. Although a proportion of violent incidents are not reported to the police, these data remain the most detailed and accurate records of violent incidents at the premises or street level.

We conducted pilot work using South Wales Police data to establish the feasibility of using police recorded incident data for the period of the proposed study, 2005–09. Approximately 42% of incidents were recorded as involving alcohol on the part of the offender or the victim. While this rate is slightly below the 50% prevalence among incidents recorded by the British Crime Survey [55] we believe that these data are of sufficient quality for our study. Furthermore, the recording of incident type and presence of injury allows the study to explore the role of outlet density for both occurrence and severity. The Police data aggregated at LSOA-level will be added to SAIL for this study.

### Proposed sample size

To investigate the magnitude of change in outlets over time, in a pilot exercise we have assessed the outlet data on licensed premises supplied by four local authorities, with a total of 273 LSOAs (mean 13 outlets). The mean five-year change for local authorities was 6% (range 5% to 46%) which exceeds the 4% change in outlets over a six-year period reported in the study of change in outlet density which gave sufficient power to detect change in a rare (suicide) outcome [34] and the −0.4% mean change in outlets over a six-year period reported in a second longitudinal study [26]. Thus we expect to have sufficient change in outlet density for the analysis.

For the primary outcome, based on data from five Welsh Health Surveys, the sample size is approximately 75 000. Because we do not yet have the data on the location of alcohol outlets by year, we do not know the distribution of changes and so cannot complete a conventional sample size calculation. However we previously carried out an unpublished analysis of data of approximately 60 000 respondents to four successive Welsh Health Surveys 2005–2008 and can draw on that experience. In similar multilevel models to those anticipated here, for the primary outcome analysis, standard errors were typically between 0.04 and 0.06, varying between model parameters. These led to narrow confidence intervals for parameter values; for example the estimated odds ratio associated with a quintile of deprivation was 0.82 with 95% confidence interval (0.76, 0.88). So with a larger sample size in this study the precision will be greater and smaller differences in outcomes will be detected.

For the other outcomes the study population is essentially the 2.4 million people living in Wales aged at least 16 years. The outcomes are not rare with approximately 13 000 alcohol-related admissions, 100 000 night time A&E attendances, and 47 000 violence against the person crimes in Wales annually. We will have very high power for detecting small differences between LSOAs at the extremes of changes in alcohol outlet density. For example there would be over 80% power for identifying a reduction from 5.4 per thousand per annum to 5 per 1000 per annum in hospital admissions between the extreme quintiles of change in outlet density. While this ignores clustering within LSOA, that effect is unlikely to be so large that the power is greatly reduced and there will be more power for detecting a larger difference. If the correction factor for clustering was two, a common finding in studies of this type, the required sample size would effectively be doubled. However with the planned sample size there would still be over 80% power for detecting a reduction from 5.4 per thousand per annum to 4.8 per thousand per annum in the admission rate for alcohol-related conditions.

### Statistical analysis

The primary analysis will be for all alcohol outlets but we will also analyse the data using outlet densities for on- and off-sales separately where available. Our analysis plan has to take into account the nature of the data available through SAIL. Data on individuals nested within households nested within anonymised LSOAs will be available. Our analysis plan is:

(1) Full descriptive statistics.

(2) Spatial models to explore the relationship between counts/rates of the four outcome measures and outlet density. The models will account for the year and will adjust for aggregated population characteristics, such as the percentages of males and of certain socio-economic groups, and the median age. There are a number of methodologies that could be adapted to calculate spatial autocorrelation, including the Bayesian approach of Besag, York and Mollié [56], the random effects models developed by Gruenewald and colleagues and used in the three Californian longitudinal studies [25,26,34] and geographically weighted regression (GWR) models [57-60]. Moreover, more complex methodologies which take into account network distances – the distance to an outlet based on road and footpath measurements – can be adapted to explore these relationships. These methodologies include network distance calculations [61], the two-step floating catchment technique [62-64], and modified GWR models [65]. The different types of spatial models make different assumptions, model spatial autocorrelation differently and analyse the data in different ways; their relative merits will be explored as part of the study.

(3) A multilevel analysis using the hierarchy outlined above. We will use multilevel ordinal models of the defined categories of consumption for the WHS data and multilevel logistic models of the odds of hospital admission and A&E attendance as a function of change in individual outlet exposure, adjusting for confounding individual variables (age, gender, socio-economic status) and LSOA deprivation quintile. This method cannot be used for the crime data as we only know the location of offence with no information on individuals. Multilevel modelling is a well-established technique, see for example Goldstein 2003 [66], and we will fit models using the standard MLwiN [67] and R software [68].

In order to address the secondary research questions:

(4) We will assess the impact on health inequalities by firstly stratifying the spatial and multilevel models using quintiles of the Welsh Index of Multiple Deprivation to examine whether the modelled associations vary with deprivation category. This method was used for postcode sectors in the Australian longitudinal analysis [29]. A further method which we will use is to model the interaction between LSOA deprivation score and change in outlet density and the two-way interaction including individual SES (using the NS-SEC) [69]. This method uses the whole dataset and the coefficients of the interaction terms give information on the effect on inequalities over time.

(5) Since the annual estimation of outlet density exposure automatically includes people who move residence, (from using the WDS within SAIL), our main models will correct for population migration. We will assess the impact of population migration by re-estimating annual outlet densities using the baseline population, repeating the models above, and comparing to the main models. Health selection bias can thus be assessed.

### Presentation and mapping of the results

An important element of the research will be mapping of the data. This will form part of the substantive investigation as well as part of the presentation and communication of the results. It is well established that mapping health and related social and economic data can provide important insights into the causal processes that affect health outcomes [70]. Global and local statistical measures of spatial clusters can be misleading without mapping [71,72], which provides an excellent method of revealing potential anomalies in the data. The research will therefore use mapping techniques in the specification and testing of the alcohol outlet density variable and the spatial model.

Mapping will also be used as part of the diagnostic tests and interpretation of the multilevel models, principally through mapping the areal residuals. This is an important element of model building [73] and can be used to refine the specification of the model as well as identify structure not accounted for by the predictors. For instance, systematic variation in the size and direction of residuals across space can be indicative of spatial autocorrelation and/or spatial heterogeneity caused by mis-specification of the model. Mapping the residuals and analysing them using spatial statistical techniques such as local indicators of spatial autocorrelation can prove invaluable in model building. Moreover, mapping will allow a visual analysis of the spatial correlation between the residuals and the location of the alcohol outlets. A clear relation, such as concentrations of large positive/negative residuals around clusters of alcohol outlets, could indicate social processes unexplained by the model and lead to further analysis.

## Discussion

This study requires a wide range of inter-disciplinary expertise, including epidemiology and public health, health informatics, medical statistics, geographical information science and health geography, city and regional planning and urban spatial analysis, systematic reviews, and violence and society alcohol research. The study is overseen by an inter-disciplinary and multi-agency study steering committee (SSC), constituted to include the principal investigator and one co-applicant from Cardiff and Swansea University, the funder, the study sponsor (Cardiff University), with a representative from South Wales Police, the Cardiff Community Safety Partnership, the Clinical Research Collaboration-Cymru ‘Involving People’ network and experts in the field of alcohol and violence research, and alcohol policy.

### Research governance and ethical arrangements

The information governance requirements for use of the Secure Anonymised Information Linkage (SAIL) dataset in this study have been approved by the Independent Information Governance Review Panel (IGRP), with membership comprised of senior representatives from the British Medical Association (BMA), the National Research Ethics Service (NRES), Public Health Wales, NHS Wales Informatics Service (NWIS), and Involving People.

Participant consent is not required because all the SAIL study outcome data on patients (hospital admissions and A&E attendances) will be anonymised before they are incorporated into the SAIL databank. As the SAIL databank is fully anonymised, it does not fall into the remit of the National Information Governance Board who provide section 251 (formerly section 60) exemption to use identifiable data without consent.

The anonymised Welsh Health Survey dataset is supplied under a formal Data Access Agreement between Cardiff University and the Welsh Government. Welsh Health Survey data do not have permission from the Data Owner (Welsh Government) to link into SAIL at individual-level but can be linked, using data aggregated by LSOA, to outlet density exposures. Police data are also supplied under Data Access Agreements between each Police Force in Wales and Cardiff University and can also only be linked at aggregate LSOA-level.

The Chairman of the Research Ethics Committee for Wales has confirmed in writing that this study does not require ethical approval under NHS research governance arrangements.

### Service users/public involvement

The scope for patient involvement is limited as we will carry out a secondary analysis of anonymously record-linked datasets. The primary vehicle in Wales for public engagement, with whom we will work closely, is the Involving People network, which aims to provide input into the strategy, development and implementation of health and social care research in Wales.

### Dissemination

A dissemination strategy is planned to include policy makers in national and local government and the research community. Maps will also form an important part of the presentation and communication of the results to different audiences. Visual information and maps are able to present a large amount of complex information in an efficient manner and will be able to visually demonstrate any spatial correlations found between alcohol outlets and the study outcomes.

## Competing interests

The author(s) declare that they have no competing interests.

## Authors’ contributions

DF conceived the study and is principal investigator. All authors made a substantial contribution to the design of the study. AW carried out the literature searches and DF, JW, FD and JM critically appraised the research papers. SR, CW, SL, NS, SO and RF designed the methods of estimating outlet density and the geographical analysis. FD and DF designed the statistical analysis. RL and SR led the use of SAIL methodology. IB and VS designed the use of Police data. DF wrote the manuscript and all authors contributed to critical revision of the final version. All authors read and approved the final manuscript and the decision to submit the manuscript for publication.

## Pre-publication history

The pre-publication history for this paper can be accessed here:

http://www.biomedcentral.com/1471-2458/12/428/prepub

## References

[B1] Leon DA McCambridge J Liver cirrhosis mortality rates in Britain from 1950 to 2002: an analysis of routine data Lancet 2006 367 52 56 10.1016/S0140-6736(06)67924-5 16399153

[B2] International Agency for Research on Cancer (IARC) Alcohol Drinking. IARC Monographs on the Evaluation of Carcinogenic Risks to Humans 44 1998 IARC, Lyon PMC7681469 1683674

[B3] Narod SA Alcohol and risk of breast cancer (editorial) JAMA 2011 306 17 1920 1921 10.1001/jama.2011.1589 22045772

[B4] Klatsky AL Gunderson E Alcohol and hypertension: a review J Am Soc Hypertens 2008 2 5 307 317 10.1016/j.jash.2008.03.010 20409912

[B5] Reynolds K Lewis LB Nolen JDL Kinney GL Sathya B He J Alcohol Consumption and Risk of Stroke: A Meta-analysis JAMA 2003 289 5 579 588 10.1001/jama.289.5.579 12578491

[B6] Parker RN Alcohol, homicide, and cultural context: a cross-national analysis of gender-specific homicide victimization Homicide Studies 1998 2 1 6 10.1177/1088767998002001002

[B7] Ramstedt M Alcohol and suicide in 14 European countries Addiction 2001 96 59 75 10.1080/09652140020021189 11228079

[B8] del Rio MC Gomez J Sancho M Alvarez F Alcohol, illicit drugs and medicinal drugs in fatally injured drivers in Spain between 1991 and 2000 Forensic Sci Int 2002 127 63 70 10.1016/S0379-0738(02)00116-0 12098527

[B9] Abramsky T Watts CH Garcia-Moreno C Devries K Kiss L Ellsberg M Jansen HAFM Heise L What factors are associated with recent intimate partner violence? Findings from the WHO multi-country study on women’s health and domestic violence BMC Publ Health 2011 11 109 10.1186/1471-2458-11-109 PMC3049145 21324186

[B10] Sivarajasingam V Morgan P Matthews K Shepherd JP Walker R Trends in violence in England and Wales 2000–2004: An Accident and Emergency Perspective Injury 2009 40 820 825 10.1016/j.injury.2008.08.017 19524898

[B11] McAlaney J McMahon J Establishing rates of binge drinking in the UK : Anomalies in the data Alcohol Alcohol 2006 41 4 355 357 16624839 10.1093/alcalc/agl025

[B12] Smith L Foxcroft D Drinking in the UK. An exploration of trends 2009 Joseph Rowntree Foundation, York

[B13] Hayward R Sharp C Young people, crime and anti-social behaviour: findings from the 2003 Crime and Justice Survey Home Office Research Findings 2005 245 1473 8406

[B14] Richardson A Budd T Home Office Research Studies 263. Alcohol, Crime and Disorder: A Study of Young Adults 2003 Home Office, London

[B15] Flatley J Kershaw C Smith K Chaplin R Moon, D: Crime in England and Wales 2009/10 2010 Home Office, London

[B16] Pletcher MJ Varosy P Kiefe CI Lewis CE Sidney S Hulley SB Alcohol Consumption, Binge Drinking, and Early Coronary Calcification: Findings from the Coronary Artery Risk Development in Young Adults (CARDIA) Study Am J Epidemiol 2005 161 5 423 433 10.1093/aje/kwi062 15718478

[B17] Bagnardi V Zatonski W Scotti L La Vecchia C Corrao G Does drinking pattern modify the effect of alcohol on the risk of coronary heart disease? Evidence from a meta-analysis J Epidemiol Community Health 2008 62 7 615 619 10.1136/jech.2007.065607 18559444

[B18] Robinson S Harris H Smoking and drinking among adults, 2009. A report on the 2009 General Lifestyle Survey [Internet]. Office for National Statistics 2011 http://www.ons.gov.uk/ons/rel/ghs/general-lifestyle-survey/2009-report/index.html

[B19] British Medical Association Alcohol misuse: tackling the UK epidemic 2008 BMA, London

[B20] Babor TF Tackling alcohol misuse in the UK (editorial) BMJ 2008 336 455 10.1136/bmj.39496.556435.80 18296458 PMC2258338

[B21] Popova S Giesbrecht N Bekmuradov D Patra J Hours and days of sale and density of alcohol outlets: Impacts on alcohol consumption and damage: A systematic review Alcohol Alcohol 2009 44 5 500 516 19734159 10.1093/alcalc/agp054

[B22] Tatlow JR Clapp JD Hohman MM The relationship between the geographic density of alcohol outlets and alcohol-related hospital admissions in San Diego County J Epidemiol Community Health 2000 25 1 79 88 10.1023/a:1005145018975 10706211

[B23] Theall KP Scribner R Cohen D Bluthenthal RN Schonlau M Lynch S Farley TA The neighborhood alcohol environment and alcohol-related morbidity Alcohol Alcohol 2009 44 5 491 499 19671569 10.1093/alcalc/agp042 PMC2800249

[B24] Campbell CA Hahn RA Elder R Brewer R Chattopadhyay S Fielding J Naimi TS Toomey T Lawrence B Middleton JC Guide to Community Preventive Services: The Effectiveness of Limiting Alcohol Outlet Density as a Means of Reducing Excessive Alcohol Consumption and Alcohol-Related Harms Am J Prev Med 2009 6 556 569 19944925 10.1016/j.amepre.2009.09.028

[B25] Gruenewald PJ Remer L Changes in outlet densities affect violence rates Alcohol Clin Exp Res 2006 30 7 1184 1193 10.1111/j.1530-0277.2006.00141.x 16792566

[B26] Treno AJ Johnson FW Remer LG Gruenewald PJ The impact of outlet densities on alcohol-related crashes: A spatial panel approach Accid Anal Prev 2007 39 5 894 901 10.1016/j.aap.2006.12.011 17275773

[B27] Lapham SC Gruenwald PJ Remer L Layne L New Mexico's 1998 drive-up liquor window closure: Effect on alcohol-involved crashes Addiction 2004 99 5 598 606 10.1111/j.1360-0443.2004.00708.x 15078234

[B28] Norstrom T Outlet density and criminal violence in Norway, 1960–1995 J Stud Alcohol 2000 61 6 907 911 11188497 10.15288/jsa.2000.61.907

[B29] Livingston M A longitudinal analysis of alcohol outlet density and assault Alcohol Clin Exp Res 2008 32 6 1074 1079 10.1111/j.1530-0277.2008.00669.x 18445114

[B30] Yu QZ Scribner R Carlin B Theall K Simonsen N Ghosh-Dastidar B Cohen D Mason K Multilevel spatio-temporal dual changepoint models for relating alcohol outlet destruction and changes in neighbourhood rates of assaultive violence Geospat Health 2008 2 2 161 172 18686265 10.4081/gh.2008.240 PMC2995332

[B31] Yu QZ Li B Scribner RA Hierarchical additive modeling of nonlinear association with spatial correlations-An application to relate alcohol outlet density and neighborhood assault rates Stat Med 2009 28 14 1896 1912 10.1002/sim.3600 19402025

[B32] Cohen DA Ghosh-Dastidar B Scribner R Miu A Scott M Robinson P Farley TA Bluthenthal RN Brown-Taylor D Alcohol outlets, gonorrhea, and the Los Angeles civil unrest: A longitudinal analysis Soc Sci Med 2006 62 12 3062 3071 10.1016/j.socscimed.2005.11.060 16423436 PMC2040035

[B33] Theall KP Scribner R Ghosh-Dastidar B Cohen D Mason K Simonsen N Neighbourhood alcohol availability and gonorrhea rates: Impact of social capital Geospat Health 2009 3 2 241 255 19440966 10.4081/gh.2009.224 PMC6167931

[B34] Johnson FW Gruenewald PJ Remer LG Suicide and Alcohol: Do Outlets Play a Role? Alcohol Clin Exp Res 2009 33 12 2124 2133 10.1111/j.1530-0277.2009.01052.x 19764933 PMC3071536

[B35] Huckle T Huakau J Sweetsur P Huisman O Casswell S Density of alcohol outlets and teenage drinking: Living in an alcogenic environment is associated with higher consumption in a metropolitan setting Addiction 2008 103 10 1614 1621 10.1111/j.1360-0443.2008.02318.x 18821871

[B36] Pollack CE Cubbin C Ahn D Winkleby M Neighbourhood deprivation and alcohol consumption: Does the availability of alcohol play a role? Int J Epidemiol 2005 34 4 772 780 10.1093/ije/dyi026 15737966

[B37] Ellaway A Macdonald L Forsyth A Macintyre S The socio-spatial distribution of alcohol outlets in Glasgow city Health Place 2010 16 167 172 10.1016/j.healthplace.2009.08.007 19775927

[B38] Norman P Boyle P Rees P Selective migration, health and deprivation: a longitudinal analysis Soc Sci Med 2005 60 12 2755 2771 10.1016/j.socscimed.2004.11.008 15820585

[B39] Connolly S O'Reilly D Rosato M Increasing inequalities in health: is it an artefact caused by the selective movement of people? Soc Sci Med 2007 64 10 2008 2015 10.1016/j.socscimed.2007.02.021 17379374

[B40] Petticrew M Cummins S Ferrell C Findlay A Higgins C Hoy C Kearns A Sparks L Natural experiments: an underused tool for public health? Public Health 2005 119 9 751 757 10.1016/j.puhe.2004.11.008 15913681

[B41] Doyle-Francis M Sadler K Kingdon A Roberts C Walters L Welsh Health Survey User Guide 2011 National Centre for Social Research, Welsh Assembly Government, http://wales.gov.uk/docs/statistics/2011/110127surveyguideen.pdf

[B42] Department of Health, Home Office, Department for Education and Skills and Department for Culture, Media and Sport Safe. Sensible. Social. The next steps in the National Alcohol Strategy 2007 DH Publications, London http://www.dh.gov.uk/en/Publicationsandstatistics/Publications/PublicationsPolicyAndGuidance/DH_075218

[B43] Gartner A Cosh H Gibbon R Lester N A profile of alcohol and health in Wales National Public Health Service for Wales/Wales Centre for Health; 2009. ISBN: 0-9545544-9-3, http://www.wales.nhs.uk/sites3/Documents/568/Alcohol%20and%20Health%20in%20Wales_WebFinal_E.pdf

[B44] Ford DV Jones KH Verplancke JP Lyons RA John G Brown G Brooks CJ Thompson S Bodger O Couch T Leake K The SAIL Databank: building a national architecture for e-health research and evaluation BMC Health Serv Res 2009 9 1 157 10.1186/1472-6963-9-157 19732426 PMC2744675

[B45] Lyons RA Jones KH John G Brooks CJ Verplancke JP Ford DV Brown G Leake K The SAIL databank: linking multiple health and social care datasets BMC Med Inform Decis Mak 2009 9 3 24 19149883 10.1186/1472-6947-9-3 PMC2648953

[B46] Office for National Statistics Super Output Areas (SOAs) http://www.ons.gov.uk/ons/guide-method/geography/beginner-s-guide/census/super-output-areas--soas-/index.html

[B47] Rodgers SE Lyons RA Dsilva R Jones KH Brooks CJ Ford DV John G Verplancke JP Residential Anonymous Linking Fields (RALFs): A Novel Information Infrastructure to Study the Interaction between the Environment and Individuals’ Health J Public Health (Oxf) 2009 31 4 582 588 10.1093/pubmed/fdp041 19447812

[B48] Ordnance Survey OS® AddressBase® Premium - CSV. Technical specification. © Crown copyright 2012 http://www.ordnancesurvey.co.uk/oswebsite/docs/technical-specifications/addressbase-premium-technical-specification-csv.pdf

[B49] Ordnance Survey OS® MasterMap® Integrated Transportation Network™ Layer technical specification. © Crown copyright 2011 http://www.ordnancesurvey.co.uk/oswebsite/support/products/os-mastermap/itn-layer-technical-specification/index.html

[B50] Ordnance Survey OS® Points of Interest database - user guide and technical specification. © Crown copyright 2010 http://www.ordnancesurvey.co.uk/oswebsite/docs/user-guides/points-of-interest-user-guide.pdf

[B51] Office for National Statistics Alcohol-related deaths in the United Kingdom 2010 http://www.ons.gov.uk/ons/rel/subnational-health4/alcohol-related-deaths-in-the-united-kingdom/2010/stb-alcohol-related-deaths.html#tab-Definition

[B52] Alcohol-Related ICD Codes http://apps.nccd.cdc.gov/DACH_ARDI/Info/ICDCodes.aspx

[B53] Local Government Data Unit – Wales Welsh Index of Multiple Deprivation 2005. Summary Report. LGDU 2005 http://wales.gov.uk/topics/statistics/publications/publication-archive/wimd2005revised/?lang=en

[B54] Moore SC Brennan I Murphy S Predicting and measuring premises-level harm in the night-time economy Alcohol Alcohol 2011 46 3 357 363 21355007 10.1093/alcalc/agr011

[B55] Flatley J Kershaw C Smith K Chaplin R Moon D Crime in England and Wales 2009/10 2010 Home Office, London

[B56] Besag J York J Mollié M Bayesian image restoration with two applications in spatial statistics Ann Inst Stat Math 1991 43 1 59 10.1007/BF00116466

[B57] Brunsdon C Fotheringham AS Charlton ME Geographically weighted regression- modelling spatial non-stationarity J R Stat Soc Ser D (The Statistician) 1998 47 3 431 443 10.1111/1467-9884.00145

[B58] Fotheringham AS Brunsdon C Charlton ME Geographically weighted regression: a natural evolution of the expansion method for spatial data analysis Environ Plan A 1998 30 11 1905 1927 10.1068/a301905

[B59] Fotheringham AS Charlton ME Brunsdon C Geographically Weighted Regression: the analysis of spatially varying relationships 2002 Wiley, Chichester, West Sussex; Hoboken, NJ

[B60] Ward MD Gleditsch KS Spatial Regression Models 2008 Sage Publications, Thousand Oaks

[B61] Rodgers SE Demmler JC Dsilva R Lyons RA Protecting health data privacy while using residence-based environment and demographic data Health Place 2012 18 2 209 217 10.1016/j.healthplace.2011.09.006 21996431

[B62] Luo W Using a GIS-based floating catchment method to assess areas with shortage of physician Health Place 2004 10 1 11 10.1016/S1353-8292(02)00067-9 14637284

[B63] Luo W Qi Y An enhanced two-step floating catchment area (E2SFCA) method for measuring spatial accessibility to primary care physicians Health Place 2009 15 1100 1107 10.1016/j.healthplace.2009.06.002 19576837

[B64] Langford M Fry R Higgs G Measuring transit system accessibility using a modified two-step floating catchment technique Int J Geogr Inf Sci 2012 26 2 193 214 10.1080/13658816.2011.574140

[B65] Lu B Charlton M Fotheringhama AS Geographically Weighted Regression Using a Non-Euclidean Distance Metric with a Study on London House Price Data Procedia Environmental Sciences 2011 7 92 97

[B66] Goldstein H Multilevel Statistical Models 2003 3 Edward Arnold, London

[B67] Rasbash J Steele F Browne W Goldstein H A User’s Guide to MlwiN version 2.10 2009 University of Bristol: Centre for Multilevel Modelling,

[B68] The R Project for Statistical Computinghttp://www.r-project.org/

[B69] Office for National Statistics The National Statistics Socio-economic Classification (NS-SEC) http://www.ons.gov.uk/ons/guide-method/classifications/archived-standard-classifications/ns-sec/index.html

[B70] Elliott P Wakefield JC Best NG Briggs DJ Spatial Epidemiology: Methods and Applications 2001 Oxford University Press, Oxford

[B71] Anselin L Local indicators of spatial association-LISA Geogr Anal 1995 27 2 93 115

[B72] Anselin L How (not) to Lie with Spatial Statistics Am J Prev Med 2006 30 2 S3 S6 10.1016/j.amepre.2005.09.015 16458788

[B73] Belsley DA Kuh E Welsch RE Regression diagnostics, identifying influential data and sources of collinearity 2004 Wiley, New Jersey

